# Affordable Care Act and healthcare delivery: A comparison of California and Florida hospitals and emergency departments

**DOI:** 10.1371/journal.pone.0182346

**Published:** 2017-08-03

**Authors:** Monique T. Barakat, Aditi Mithal, Robert J. Huang, Alka Mithal, Amrita Sehgal, Subhas Banerjee, Gurkirpal Singh

**Affiliations:** 1 Division of Gastroenterology and Hepatology, Stanford University Medical Center, Stanford, California, United States of America; 2 Institute of Clinical Outcomes Research and Education (ICORE), Woodside, California, United States of America; 3 Wharton School of the University of Pennsylvania, Philadelphia, Pennsylvania, United States of America; Old Dominion University, UNITED STATES

## Abstract

**Importance:**

The Affordable Care Act (ACA) has expanded access to health insurance for millions of Americans, but the impact of Medicaid expansion on healthcare delivery and utilization remains uncertain.

**Objective:**

To determine the early impact of the Medicaid expansion component of ACA on hospital and ED utilization in California, a state that implemented the Medicaid expansion component of ACA and Florida, a state that did not.

**Design:**

Analyze all ED encounters and hospitalizations in California and Florida from 2009 to 2014 and evaluate trends by payer and diagnostic category. Data were collected from State Inpatient Databases, State Emergency Department Databases and the California Office of Statewide Health Planning and Development.

**Setting:**

Hospital and ED encounters.

**Participants:**

Population-based study of California and Florida state residents.

**Exposure:**

Implementation of Medicaid expansion component of ACA in California in 2014.

**Main outcomes or measures:**

Changes in ED visits and hospitalizations by payer, percentage of patients hospitalized after an ED encounter, top diagnostic categories for ED and hospital encounters.

**Results:**

In California, Medicaid ED visits increased 33% after Medicaid expansion implementation and self-pay visits decreased by 25% compared with a 5.7% increase in the rate of Medicaid patient ED visits and a 5.1% decrease in rate of self-pay patient visits in Florida. In addition, California experienced a 15.4% increase in Medicaid inpatient stays and a 25% decrease in self pay stays. Trends in the percentage of patients admitted to the hospital from the ED were notable; a 5.4% decrease in hospital admissions originating from the ED in California, and a 2.1% decrease in Florida from 2013 to 2014.

**Conclusions and relevance:**

We observed a significant shift in payer for ED visits and hospitalizations after Medicaid expansion in California without a significant change in top diagnoses or overall rate of these ED visits and hospitalizations. There appears to be a shift in reimbursement burden from patients and hospitals to the government without a dramatic shift in patterns of ED or hospital utilization.

## Introduction

In January, 2014, the Patient Protection and Affordable Care Act [commonly known as the Affordable Care Act (ACA)] included a Medicaid expansion component which extended access to health insurance coverage to over 20 million previously uninsured individuals, with a resultant 21% increase in Medicaid enrollment [1, 2]. The primary driver of this sharp increase was expansion of Medicaid eligibility to adults under 65 years of age earning up to 138% of the federal poverty level. However, a 2012 Supreme court ruling allowed states to opt out of Medicaid expansion. Currently 32 states and the District of Columbia have elected to expand Medicaid, whereas 19 states have not [1]. Following Medicaid expansion in California, there was an estimated 45% reduction in the uninsured population, with 1.3 million individuals gaining Medicaid coverage in January, 2014 and an additional 2.1 million individuals gaining Medicaid coverage by December, 2014 [3, 4].

There is considerable controversy regarding the impact of Medicaid expansion on health care costs, hospital readmission rates and quality of care [1, 5–7]. There are few data evaluating the impact of Medicaid expansion on Emergency Department (ED) utilization. Assessment of ED utilization after Medicaid expansion is important, because the ED is a suboptimal environment for delivery of non-urgent care. ED visits for primary care treatable conditions are costly, lead to ED overcrowding, and result in fragmented care. Furthermore, in the ED environment, care is often delivered without knowledge of a patient’s comprehensive medical history and comorbidities. Prior to full Medicaid expansion, annual Emergency Department (ED) visit rates in the United States were increasing more than would be expected from population growth,[8] with a rising proportion of Medicaid or uninsured patients [9, 10]. One aim of the ACA was to facilitate better access to primary care. Providing Medicaid coverage to the uninsured as a part of Medicaid expansion was an important component of this plan, however, Medicaid patients are among the highest utilizers of the ED [8]. Determining the impact of Medicaid expansion on ED utilization and hospitalizations is therefore a matter of great interest.

In this study we evaluate all ED visits and the top ED visit diagnoses, the percentage of patients admitted to hospitals from the ED, all hospitalizations and top hospitalization diagnoses over the five-year period leading up to Medicaid expansion and the one year following Medicaid expansion, and compare results between a Medicaid expansion state (California) and a non-expansion state (Florida).

## Methods

### Data sets

We evaluated all states for which data were available at the time of the analysis. Of these, California and Florida are largest and most demographically similar, with substantial immigrant populations. We analyzed all-cause ED encounters and hospitalizations in the states of California and Florida between the years 2009 and 2014. Data from Florida from 2009 to 2014 and from California from 2009 to 2011, were collected from the State Inpatient Databases (SID) and the State Emergency Department Databases (SEDD) maintained by the Agency for Healthcare Research and Quality (AHRQ), Healthcare Cost and Utilization Project (HCUP) [11]. The SID includes state inpatient discharge records encompassing all patients, regardless of payer, providing a unique view of inpatient care in a state over time. Data elements include resource-use information that is included in a typical discharge abstract, with safeguards to protect the privacy of individual patients, physicians, and hospitals [11]. The SEDD captures ED visits at hospital-affiliated EDs that do not result in hospitalization. If an ED visit does result in hospitalization, information regarding the encounter is then reported through the SID. Data reported from SEDD includes a core set of clinical and non-clinical information for all patients regardless of payer [11]. As the SID and SEDD are not available for California from 2012 to 2014, ED and hospital utilization data were collected from the California Office of Statewide Health Planning and Development (OSHPD), public use files Patient Discharge Data (PDD) and Emergency Department Data (EDD) [12]. OSHPD data are identical to SID and SEDD data, as OSHPD is the source for these data. OSHPD collects data for multiple types of health care facilities in California, including hospitals and emergency departments. An inpatient discharge or emergency department visit record is submitted to OSHPD each time a patient is treated in a licensed facility in California. Data from OSHPD and other state agencies are used to generate the HCUP data sets. As a sensitivity analysis, total ED visits and hospitalizations from 2009 to 2011 were analyzed with both the OSHPD and HCUP data, with consistent results.

### Patient cohort

We evaluated all ED encounters and hospitalizations from the states of California and Florida during the study period. Of note, due to the nature of databases used, ED visits and hospitalizations analyzed include visits to facilities in California and Florida and these may be visits for individuals who do not reside in California or Florida but are visiting the area. The total number of ED encounters, total number of hospital discharges, and total number of ED encounters resulting in hospital admission were evaluated. Each ED encounter or hospitalization was classified by payer and clinical diagnoses grouped using Clinical Classification Software (CCS) multi-level diagnosis. The CCS is a tool developed by the AHRQ to cluster *International Classification of Diseases*, *9th Revision*, *Clinical Modification* (ICD-9-CM) diagnoses and procedures into a manageable number of clinically meaningful categories [13].

### Analyses

The Medicaid expansion provisions of the ACA became effective on January 1, 2014. The pre-Medicaid expansion period was defined as prior to this date, and the post-Medicaid expansion period was defined as after this date. Per capita rates of ED encounters and hospitalizations were calculated for California and Florida for each year. State population data were obtained from the U.S. Census Bureau Bridged-Race population estimates [14]. The per capita rates of ED encounters and hospitalizations were then stratified according to primary payer. Rates of ED visits resulting in hospitalization were calculated for each year, and stratified by payer. Per capita rates of ED encounters and hospitalizations were further stratified by the most common CCS multi-level diagnosis categories for each state and year.

Linear regression analysis was performed on 2009–2013 data to compare the predicted relative to actual rate of Medicaid ED visits in 2014. All analyses were performed using SAS version 9.4 (SAS Institute, Inc., Cary, NC) and Microsoft Excel 2016.

## Results

### Demographics

Population demographics over the study period are depicted in Table 1. The overall US population was 306,771,529 in 2009 and 318,907,401 in 2014 (3.96% increase). The population of California was 36,961,229 in 2009 and 38,792,291 in 2014 (4.95% increased) and the population of Florida was 18,652,644 in 2009 and 19,905,569 in 2014 (6.72% increased) (Table 1). California and Florida represented 12.05% and 6.08% of the US population in 2009 and 12.16% and 6.24% of the US population in 2014, respectively. There are significant differences in age, race and ethnicity between the two states. Florida has a higher proportion of people aged 65 years of age and older. In 2009, 17.19% of Florida’s population was aged ≥ 65 years of age and older, compared to 11.46% of the California population and 12.92% of the US population. An increased proportion of all populations were 65 and older in 2014: 19.05% in Florida, 12.85% in California, and 14.49% in the US. In 2009 and 2014, Hispanics comprised 37.2% and 38.6% of California’s total population, compared with 22.2% and 24.1% of Florida’s population and 16.1% and 17.4% of the US population, respectively. In 2009 and 2014, Black individuals comprised 16.8% and 17.3% of the Florida population, compared with only 7.3% and 7.2% of the California population and 13.5% and 13.9% of the US population, respectively.

**Table 1:** Total population, racial/ethnic.

Comparison of US, Florida and California Demographics in 2009 and 2014												
Percentage of population by age and race/ethnicity												
	2009						2014					
	US		CA		FL		US		CA		FL	
	Numbers	%	Numbers	%	Numbers	%	Numbers	%	Numbers	%	Numbers	%
Total	306,771,529		36,961,229		18,652,644		318,907,401		38,792,291		19,905,569	
0–18 years	78,668,801	25.6	9,872,723	26.7	4,252,850	22.8	77,827,101	24.4	9,678,170	24.9	4,290,642	21.6
19–25 years	30,200,314	9.8	3,853,022	10.4	1,716,027	9.2	31,767,407	10.0	4,063,772	10.5	1,818,949	9.1
26–34 years	36,518,740	11.9	4,730,660	12.8	2,028,143	10.9	39,035,791	12.2	5,123,045	13.2	2,263,011	11.4
35–44 years	41,487,811	13.5	5,214,338	14.1	2,460,117	13.2	40,534,422	12.7	5,176,950	13.3	2,427,515	12.2
45–54 years	44,867,088	14.6	5,220,443	14.1	2,715,193	14.6	43,464,825	13.6	5,243,365	13.5	2,739,674	13.8
55–64 years	35,405,600	11.5	3,905,550	10.6	2,274,599	12.2	40,076,125	12.6	4,520,695	11.7	2,574,733	12.9
Age 65+	39,623,175	12.9	4,164,493	11.3	3,205,715	17.2	46,201,730	14.5	4,986,294	12.9	3,791,045	19.0
White	199,993,079	65.2	15,538,954	42.0	11,060,801	59.3	201,025,357	63.0	15,432,084	39.8	11,260,915	56.6
Black	39,104,815	12.7	2,355,669	6.4	2,917,176	15.6	41,322,154	13.0	2,420,088	6.2	3,194,110	16.0
Asian	15,793,995	5.1	5,097,609	13.8	496,184	2.7	18,498,178	5.8	5,757,924	14.8	593,219	3.0
American Indian	2,552,151	0.8	209,130	0.6	54,176	0.3	2,666,544	0.8	209,888	0.5	57,947	0.3
Hispanics all races	49,327,489	16.1	13,759,867	37.2	4,124,307	22.1	55,395,168	17.4	14,972,307	38.6	4,799,378	24.1

United States Department of Health and Human Services (US DHHS), Centers for Disease Control and Prevention (CDC), National Center for Health Statistics (NCHS), Bridged-Race Population Estimates, United States July 1st resident population by state, county, age, sex, bridged-race, and Hispanic origin. Compiled from 1990–1999 bridged-race intercensal population estimates (released by NCHS on 7/26/2004); revised bridged-race 2000–2009 intercensal population estimates (released by NCHS on 10/26/2012); and bridged-race Vintage 2015 (2010–2015) postcensal population estimates (released by NCHS on 6/28/2016). Available on CDC WONDER Online Database. Accessed at http://wonder.cdc.gov/bridged-race-v2015.html on Aug 5, 2016

### ED utilization

**Fig 1:**
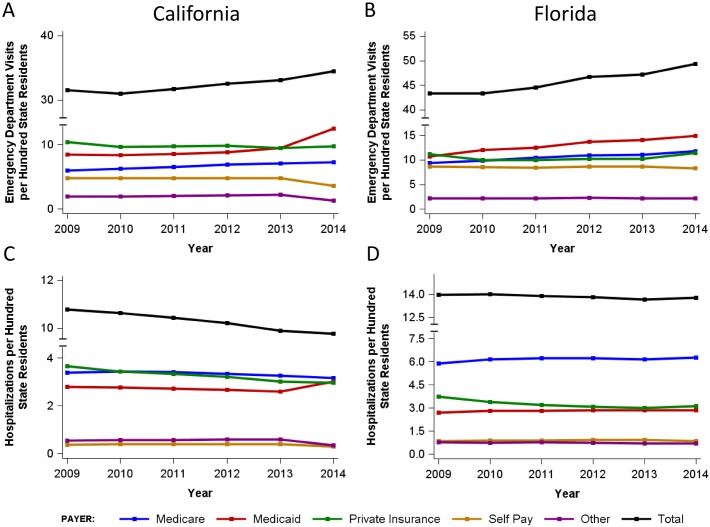
Emergency department utilization in California (A) and Florida (B) between 2009 and 2014 by payer, expressed as annual visits per 100 state residents. Hospital utilization in California (C) and Florida (D) between 2009 and 2014 by payer, expressed as annual hospitalizations per 100 state residents. See S1 Table for corresponding data tables.

**Fig 2:**
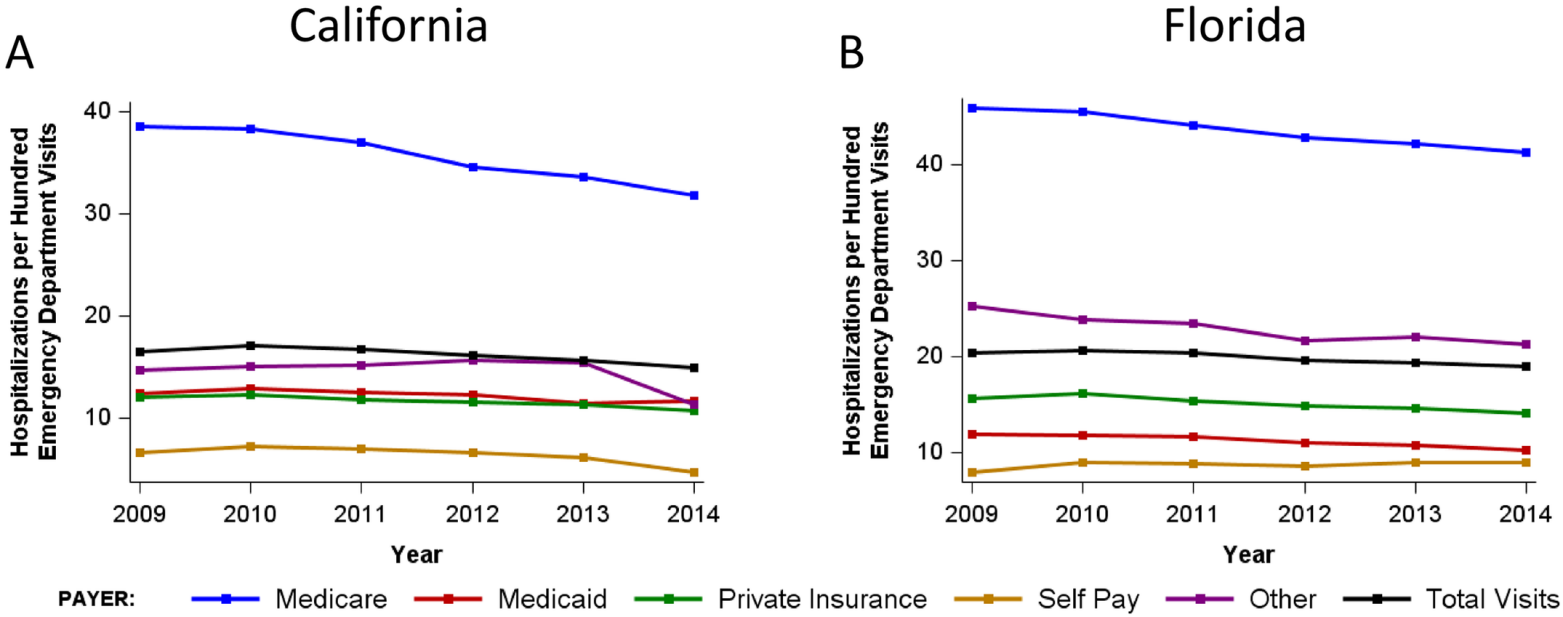
**(A)** Predicted vs. Actual Emergency Department visits in Medicaid enrollees in California (per 100 state residents). In 2014, the actual rate was 32.2% higher than predicted based on 2009–2013 data (p<0.0001). **(B)** Florida Medicaid ED visit rates did not differ from those predicted based on 2009–2013 data.

Prior to Medicaid expansion, Medicaid patient ED visits in California increased from 8.4 per 100 residents in 2009 to 9.4 per 100 residents in 2013 (11.9% increase over 5 years). Over the same period of time, Medicaid patient ED visits in Florida increased from 10.7 to 14.1 per 100 residents (31.8% increase over 5 years, S1 Table, Fig 1B). The increase in Medicaid ED visits in California in 2014 was significantly greater than predicted based on linear regression analysis of 2009–2013 data (Fig 2A), with the actual rates being 32.2% higher than predicted (p<0.0001). In contrast, Florida Medicaid ED visit rates in 2014 closely followed those predicted from 2009–2013 data (Fig 2B). After Medicaid expansion in 2014, California Medicaid patient ED visits increased to 12.5 per 100 residents—a 33% increase in the rate of Medicaid patient ED visits (S1 Table, Fig 1A) over a single year. From 2013 to 2014, a 25% decrease in self-pay patient visits was also observed in California (S1 Table, Fig 1A). In contrast, in Florida, only a 5.7% increase in Medicaid patient ED visits was noted between 2013 and 2014, with a 5.1% decrease in rate of self-pay patient visits and an 11.7% increase in the rate of private insurance patient visits in 2014 relative to 2013 (S1 Table, Fig 1B).

The total number of ED visits in California increased from 31.6 per 100 residents in 2009 to 33.1 per 100 residents in 2013 and further increased to 34.5 per 100 residents in 2014. Overall ED visits increased by 4.2% in California from 2013 to 2014, compared to an average increase of around 2% over the previous 3 years. (S1 Table, Fig 1A and 1B).

### Hospitalizations

**Fig 3:**
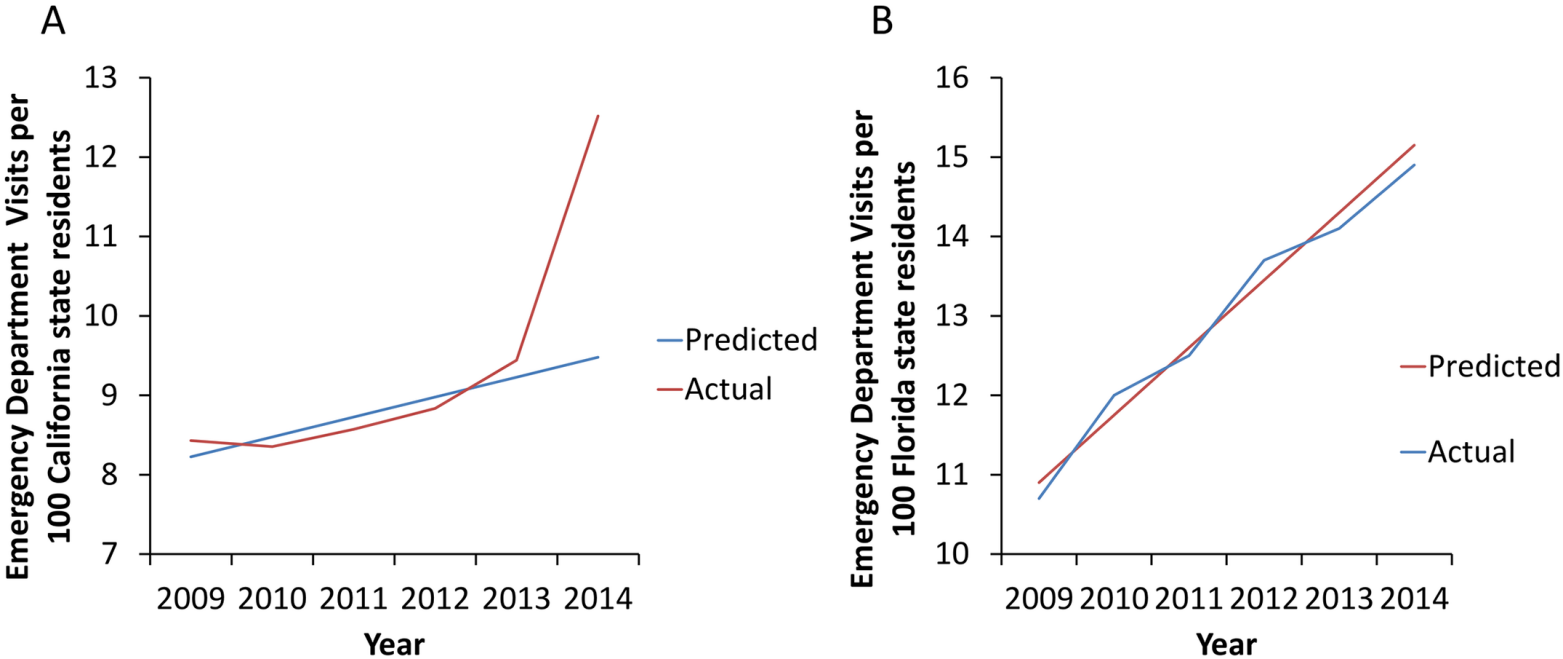
Rate of hospitalization California (A) and Florida (B) between 2009 and 2014 by payer, expressed hospitalizations per 100 emergency department visits. See S2 Table for corresponding data tables.

In the five years preceding Medicaid expansion, Medicaid hospitalizations in California decreased from 2.8 per 100 residents in 2009 to 2.6 per 100 residents in 2013 (Fig 3A and 3B, S1 Table). From 2013 to 2014, overall hospitalizations remained stable (9.9 per 100 in 2013 and 9.8 per 100 in 2014), but California hospitalization trends by payer demonstrated a 15.4% increase in the rate of inpatient stays for Medicaid patients and a 25% decline in the rate of self-pay hospitalizations (Fig 3A and 3B, S1 Table). Hospitalizations in Florida increased from 2.7 to 2.9 per 100 residents from 2009–2013 and remained stable at 2.9 per 100 residents from 2013 to 2014. Trends for other payers, including Medicare, private insurance and self-pay remained stable from 2013–2014 in Florida (S1 Table).

### Admissions from the ED

From 2009 to 2013, the rate of admissions from the ED decreased from 16.5 to 15.7 per 100 residents in California. A decrease in ED admissions rate from 20.3 (in 2009) to 19.3 per 100 residents (in 2013) was also observed in Florida. In 2014, the rate of admissions from the ED dropped in both California and Florida, to 14.9 and 18.9 per 100 residents, respectively. This reflects a sharper 5.4% decrease in ED admissions in California, and a 2.1% decrease in ED admissions in Florida (Fig 3A, S2 Table).

### Most common diagnostic categories

In addition to rates of ED visits and hospitalizations, we evaluated the most common presenting diagnoses for ED visits and hospitalizations for California and Florida in the five years before and one year after Medicaid expansion. S3 Table lists the top 10 most common Clinical Classification Software (CCS) categories by state and by year for both ED visits and hospitalizations. Incidence per 1,000 state residents is listed parenthetically. These CCS categories remained consistent from 2009–2014 for each state, with respiratory infections, heart disease, and sprains and strains among the top 10 conditions in each year in both states. (S3 Table)

## Discussion

Emergency departments are often viewed as a safety net for medically underserved patients with limited access to primary care. Adults with Medicaid insurance and patients without health insurance are high ED utilizers [8]. It was hoped that full implementation of the ACA in January, 2014 would provide insurance coverage to the uninsured (primarily through Medicaid expansion) and thus allow improved access to primary care physicians, thereby decreasing the demand for ED services, at least for primary care treatable conditions. However, many hypothesized that, despite the expansion of insurance coverage with the ACA, inadequate access to primary care for the newly insured (attributable to higher demand for care) combined with a reduction in financial barriers to using the ED, might actually increase demand for ED services [15, 16]. Although few data are available to support this hypothesis, an initial survey of over 2,000 ED physicians conducted by the American College of Emergency Physicians indicated that 75% believe that ED visits have increased since implementation of the ACA [17]. In 2008, Oregon's Medicaid program underwent what has been considered a randomized controlled trial of Medicaid expansion [18, 19]. While individuals with new Medicaid coverage in Oregon reported superior access to primary care, overall healthcare spending increased by approximately $1000 per patient annually, and Medicaid patients were found to use the ED 40% more frequently than other adult populations [18, 19]. Other studies have found that Medicaid patients, rather than the uninsured, account for the largest proportion of patients using EDs for non-urgent conditions [20]. These non-urgent ED visits are rarely preceded by a visit to a primary care provider [20]. On the other hand, a study of the 2010 ACA dependent coverage expansion provision, which allowed adults under 26 to retain insurance coverage under their parent’s insurance plan, noted a 0.5% decrease in the number of ED visits per 1,000 people in young adults aged 19 to 25 years, compared to 26–31 year-old adults unaffected by the provision [21].

We compared ED utilization in two states, one that implemented Medicaid expansion (California) and the other that opted out of this provision (Florida), using data from five years before and one year after Medicaid expansion to analyze trends over time. In the five years prior to Medicaid expansion, Medicaid patient ED visits in California increased by 11.9%, potentially associated with implementation of California’s Low Income Health Plan, a Medicaid-like plan which was implemented on a rolling county-by-county basis from November, 2010 to December, 2013 and ended in January, 2014 at which time enrollees in this plan were rolled into California’s Medicaid expansion program. As a frame of reference, following Medicaid expansion in California, 3.4 million individuals gained Medicaid coverage in 2014, compared with just 624,115 individuals who transitioned from the Low Income Health Plan to Medicaid in January, 2014 [3, 4].

In the five years prior to Medicaid expansion, Medicaid patient ED visits in Florida increased by 31.8%. Our data indicate small changes in overall ED visits with Medicaid expansion, but demonstrate a dramatic shift in the payer mix for these ED visits. Medicaid expansion resulted in a 33% increase in Medicaid patient ED visits and a corresponding sharp decline (25%) in self-pay patient visits in California. Over the same time period, in Florida, a non-expansion state, there was a small (5.7%) overall increase in Medicaid patient visits, correlating with the small increase in Medicaid enrollment from 2013 to 2014,[22] similar to the trend in preceding years. These shifts in the California payer profile are consistent with the expansion of Medicaid and the resultant coverage of previously uninsured and self-pay individuals as well as those previously enrolled in the Low Income Health Plan. The stability of overall ED visits may be attributable to a payer shift between two high ED utilization populations as uninsured individuals obtain Medicaid insurance. Overall hospitalization rates are similarly stable after Medicaid expansion in California, with a slight increase in total hospitalization rates in Florida over the same time period. Trends in payer mix for hospitalizations mirror those for ED utilization, but the overall increase in Medicaid patient hospitalizations in California after Medicaid expansion is lower, with a corresponding decline in self-pay and private insurance hospitalizations.

We show that the percentage of patients hospitalized after ED visits has been decreasing steadily over the past 5 years in California and Florida. An overall 5.4% decrease in the rate of ED visits resulting in hospitalization was noted in California after Medicaid expansion, while a smaller 2.1% decrease in ED visits resulting in hospitalizations was evident in Florida over the same time period. The stably higher percentage of patients hospitalized after ED visits in Florida is probably a consequence of its older population, many of whom may have multiple co-morbidities. Presenting diagnoses for ED visits did not significantly change over time (S3 Table). Prior to Medicaid expansion, there was speculation that ED visits for non-emergent conditions might increase significantly due to the relative lack of primary care access, given the sudden inundation of the healthcare system with a large number of newly insured Medicaid and other insured patients. If such a trend for increasingly non-emergent presentations to the ED had been seen, it might indicate that prior insurance coverage was inadequate and that previously uninsured individuals who now had insurance were seeking medical attention in the ED for lower acuity symptoms. Scrutiny of the top 10 conditions prompting ED visits (S3 Table) reveals no change in the top 10 conditions for which patients present to the ED after Medicaid expansion. However, the five-year trend of same-institution hospitalizations in California is notable for a more dramatic decline in the rate of admission from the ED after Medicaid expansion than would be projected, based on the rate of admission decrease over the 5 years preceding Medicaid expansion. This greater than expected decline in admission rates suggests that ED use for lower acuity conditions that do not fall within the list of top 10 ED diagnoses may have increased following Medicaid expansion.

The Medicaid population evaluated here includes both Medicaid fee-for-service (FFS) and Medicaid Managed Care patients. It would be of interest to understand the independent trends of these two Medicaid populations and how they may differ. Medicaid Managed Care involves a centralized care setting with managed care principles to minimize cost and optimize access and quality of care [23]. ED visits for patients with Medicaid Managed Care may then be more amenable to incentives to enhance primary care management of non-urgent conditions and decrease ED visits in the longer term [23]. However, it is also possible that barriers to accessing medical care under Medicaid Managed Care may result in an increase in ED visits and preventable hospitalizations [24].

A limitation of this study is that although we evaluated trends over five years before Medicaid expansion, we had only a single year’s data following Medicaid expansion. However, this transition year is particularly worthy of study, as it may help identify and highlight trends and bottlenecks that must be addressed to optimize future achievement of the stated goals of the ACA. Medicaid patients often encounter difficulty in finding primary care physicians who will accept their coverage, as Medicaid reimbursement rates are lower than Medicare reimbursement rates [25]. Medicaid pays only approximately 56% and Medicare 80%, of what private insurers typically pay. In anticipation of this issue, Medicaid reimbursement rates were federally funded to match Medicare reimbursement rates for a defined set of primary care-related services over 2013 and 2014, in an effort to enhance access to primary care for the new and growing number of Medicaid enrollees [26]. This ACA initiative improved primary care access as measured by appointment availability for Medicaid patients,[27] which might explain the overall stability of ED visit rates in California after Medicaid expansion. The reimbursement increase was transient and expired at the end of 2014 in most states including California [26]. It will be of great interest to determine the impact of withdrawal of this rate increase and the estimated 42.8% reduction in Medicaid reimbursement on primary care access and ED visits over subsequent years [28]. Whereas primary care physicians may continue to see Medicaid patients who have established care with their practices even though reimbursements decline, it is also possible that access to primary care may again diminish with a consequent increase in ED visits for Medicaid patients. The impact of withdrawal of the reimbursement rate increase can be evaluated as some states opted to use state funds to continue to support the Medicaid reimbursement increase in 2015, whereas several did not [29].

Studying the impact of ACA on health care utilization is challenging, because the process is not a randomized, controlled study. Other cost analyses have noted that slowing of the increase in health care costs may be attributable to the recent economic recession rather than Medicaid expansion [6]. Expensive therapeutic advances in health care may further modulate health care costs [6]. These variables prevent direct attribution of any changes in health care cost to Medicaid expansion and also, to a lesser extent, impacts our analysis of ED utilization and hospitalizations, as health care utilization may be modulated by economic downturns. Our analysis of the six-year trend in ED and hospital utilization provides longitudinal support and a context for interpreting changes that followed Medicaid expansion.

In summary, our study has demonstrated a significant shift in payer for ED visits and hospitalizations after Medicaid expansion in California. There were notably fewer self-pay and significantly more Medicaid patient admissions and ED visits after Medicaid expansion in California, with relatively stable overall ED and hospital utilization. This is consistent with a shift in the burden of reimbursement from patients and hospitals to the government. The stability of ED utilization and analyses of top diagnoses indicate that individuals with newly obtained Medicaid insurance coverage utilize the ED for conditions of similar seriousness as those who are self-pay or uninsured. If financial barriers had previously prevented uninsured patients with significant illness from seeking medical care, one would expect a dramatic increase in ED visits and hospitalizations when these individuals obtained Medicaid coverage. We found no evidence of such a trend, indicating either that health care access was sufficient prior to Medicaid expansion or that the temporary increase in Medicaid reimbursement rates have buffered the impact of Medicaid expansion on ED utilization.

## Supporting information

S1 Table Emergency department visits and hospitalizations per 100 state residents by state and by payer. B (bottom): total emergency department visits and hospitalizations by state and by payer.
(DOCX)

S2 Table Rate of hospitalization per 100 emergency department visits by state and by payer. Characteristics of California, Florida and the United States in 2009 and 2014.
(DOCX)

S3 Table Emergency department visits and hospitalizations sorted by the top 10 most common Clinical Classification Software (CCS) level-2 categories by state and by year. Incidence per 1,000 state residents is listed in parentheses.
(DOCX)
