# Supplementary material for: Affordable Care Act and healthcare delivery: A comparison of California and Florida hospitals and emergency departments
Source: PLoS One. 2017 Aug 3;12(8):e0182346. doi: 10.1371/journal.pone.0182346 (PMC5542622; doi:10.1371/journal.pone.0182346)
Supplement: S1 Table — B (bottom): total emergency department visits and hospitalizations by state and by payer. (DOCX) [file pone.0182346.s001.docx]

| **Emergency Department Visit and Hospitalization Trends by State and by Payer** | | | | | | | | | | | | |
| --- | --- | --- | --- | --- | --- | --- | --- | --- | --- | --- | --- | --- |
| **PAYER** | **2009** | **2010** | **2011** | **2012** | **2013** | **2014** | **2009** | **2010** | **2011** | **2012** | **2013** | **2014** |
| **California Emergency Department Visits** | | | | | | | **Florida Emergency Department Visits** | | | | | |
| **Medicare** | 6.0 | 6.2 | 6.6 | 6.9 | 7.1 | 7.3 | 9.4 | 9.9 | 10.5 | 11.0 | 11.1 | 11.8 |
| **Medicaid** | 8.4 | 8.4 | 8.6 | 8.8 | 9.4 | 12.5 | 10.7 | 12.0 | 12.5 | 13.7 | 14.1 | 14.9 |
| **Private Insurance** | 10.4 | 9.6 | 9.8 | 9.9 | 9.5 | 9.7 | 11.2 | 9.9 | 9.9 | 10.2 | 10.3 | 11.5 |
| **Self Pay** | 4.8 | 4.8 | 4.8 | 4.8 | 4.8 | 3.6 | 8.6 | 8.5 | 8.5 | 8.7 | 8.7 | 8.2 |
| **Other** | 2.0 | 2.0 | 2.0 | 2.1 | 2.2 | 1.3 | 2.1 | 2.1 | 2.2 | 2.3 | 2.2 | 2.2 |
| **Total** | 31.6 | 31.0 | 31.7 | 32.6 | 33.1 | 34.5 | 43.3 | 43.4 | 44.5 | 46.7 | 47.2 | 49.4 |
| **California Hospitalizations** | | | | | | | **Florida Hospitalizations** | | | | | |
| **Medicare** | 3.4 | 3.4 | 3.4 | 3.3 | 3.3 | 3.2 | 5.9 | 6.2 | 6.2 | 6.2 | 6.1 | 6.3 |
| **Medicaid** | 2.8 | 2.8 | 2.7 | 2.7 | 2.6 | 3.0 | 2.7 | 2.8 | 2.8 | 2.8 | 2.9 | 2.9 |
| **Private Insurance** | 3.7 | 3.5 | 3.3 | 3.2 | 3.0 | 3.0 | 3.7 | 3.4 | 3.2 | 3.1 | 3.0 | 3.1 |
| **Self Pay** | 0.4 | 0.4 | 0.4 | 0.4 | 0.4 | 0.3 | 0.9 | 0.9 | 0.9 | 0.9 | 0.9 | 0.9 |
| **Other** | 0.5 | 0.6 | 0.6 | 0.6 | 0.6 | 0.4 | 0.8 | 0.7 | 0.8 | 0.7 | 0.7 | 0.7 |
| **Total** | 10.8 | 10.6 | 10.4 | 10.2 | 9.9 | 9.8 | 14.0 | 14.0 | 13.9 | 13.8 | 13.6 | 13.8 |

**Supplementary Table 1: A(top):** Emergency Department visits and Hospitalizations per 100 state residents by state and by payer. **B (bottom):** Total Emergency Department visits and Hospitalizations by state and by payer.

| **Emergency Department Visit and Hospitalization Trends by State and by Payer** | | | | | | | | | | | | |
| --- | --- | --- | --- | --- | --- | --- | --- | --- | --- | --- | --- | --- |
| **PAYER** | **2009** | **2010** | **2011** | **2012** | **2013** | **2014** | **2009** | **2010** | **2011** | **2012** | **2013** | **2014** |
| **California Emergency Department Visits** | | | | | | | **Florida Emergency Department Visits** | | | | | |
| **Medicare** | 2,217,140 | 2,332,927 | 2,473,023 | 2,642,161 | 2,728,855 | 2,838,797 | 1,748,698 | 1,868,829 | 2,000,635 | 2,125,346 | 2,173,574 | 2,343,541 |
| **Medicaid** | 3,115,537 | 3,118,654 | 3,231,593 | 3,362,590 | 3,628,927 | 4,856,934 | 1,997,143 | 2,260,345 | 2,388,890 | 2,648,108 | 2,763,828 | 2,973,816 |
| **Private Insurance** | 3,834,213 | 3,599,506 | 3,684,832 | 3,749,350 | 3,635,666 | 3,781,370 | 2,082,951 | 1,874,506 | 1,900,283 | 1,977,731 | 2,012,948 | 2,278,893 |
| **Self-Pay** | 1,767,439 | 1,781,045 | 1,819,989 | 1,819,954 | 1,833,279 | 1,379,210 | 1,610,149 | 1,604,317 | 1,616,602 | 1,684,634 | 1,704,572 | 1,640,720 |
| **Other** | 726,611 | 738,548 | 757,436 | 796,751 | 858,150 | 501,686 | 396,534 | 400,773 | 427,295 | 436,474 | 425,740 | 441,036 |
| **Total** | 11,662,739 | 11,572,492 | 11,969,359 | 12,406,843 | 12,717,924 | 13,379,786 | 8,078,774 | 8,181,912 | 8,507,682 | 9,045,339 | 9,249,759 | 9,828,338 |
| **California Hospitalizations** | | | | | | | **Florida Hospitalizations** | | | | | |
| **Medicare** | 1256097 | 1286035 | 1285300 | 1267634 | 1257843 | 1227999 | 1101507 | 1161621 | 1192227 | 1206474 | 1203870 | 1244637 |
| **Medicaid** | 1036376 | 1035387 | 1022199 | 1013248 | 1000269 | 1167930 | 504010 | 528998 | 539651 | 550586 | 560427 | 567162 |
| **Private Insurance** | 1351040 | 1288685 | 1257356 | 1222199 | 1163669 | 1148441 | 694883 | 640866 | 608698 | 596129 | 587620 | 621590 |
| **Self-Pay** | 139984 | 150877 | 153434 | 156298 | 153481 | 113516 | 161737 | 167585 | 169702 | 177532 | 181868 | 171059 |
| **Other** | 201371 | 209681 | 214567 | 232392 | 231649 | 136373 | 144027 | 141022 | 145971 | 139799 | 139703 | 137536 |
| **Total** | 3,985,166 | 3,970,921 | 3,933,239 | 3,891,771 | 3,806,911 | 3,794,259 | 2,606,164 | 2,640,092 | 2,656,249 | 2,670,520 | 2,673,488 | 2,741,984 |
