# Supplementary material for: Affordable Care Act and healthcare delivery: A comparison of California and Florida hospitals and emergency departments
Source: PLoS One. 2017 Aug 3;12(8):e0182346. doi: 10.1371/journal.pone.0182346 (PMC5542622; doi:10.1371/journal.pone.0182346)
Supplement: S2 Table — Characteristics of California, Florida and the United States in 2009 and 2014. (DOCX) [file pone.0182346.s002.docx]

| **Emergency Department Visit and Hospitalization Trends by State and by Payer** |
| --- |

| **PAYER** | **2009** | **2010** | **2011** | **2012** | **2013** | **2014** | **2009** | **2010** | **2011** | **2012** | **2013** | **2014** |
| --- | --- | --- | --- | --- | --- | --- | --- | --- | --- | --- | --- | --- |
| **California Emergency Department Visits** | | | | | | | **Florida Emergency Department Visits** | | | | | |
| **Medicare** | 38.5 | 38.3 | 37.0 | 34.5 | 33.6 | 31.8 | 45.9 | 45.5 | 44.1 | 42.8 | 42.2 | 41.2 |
| **Medicaid** | 12.4 | 12.8 | 12.5 | 12.2 | 11.4 | 11.6 | 11.9 | 11.8 | 11.7 | 11.0 | 10.7 | 10.2 |
| **Private Insurance** | 12.0 | 12.3 | 11.8 | 11.5 | 11.3 | 10.7 | 15.6 | 16.1 | 15.4 | 14.9 | 14.6 | 14.1 |
| **Self Pay** | 6.6 | 7.1 | 7.0 | 6.6 | 6.1 | 4.6 | 7.9 | 8.9 | 8.8 | 8.6 | 8.9 | 9.0 |
| **Other** | 14.7 | 15.0 | 15.1 | 15.6 | 15.4 | 11.3 | 25.3 | 23.8 | 23.4 | 21.6 | 22.1 | 21.2 |
| **Total** | 16.5 | 17.1 | 16.7 | 16.1 | 15.7 | 14.9 | 20.3 | 20.7 | 20.3 | 19.6 | 19.3 | 18.9 |

**Supplementary Table 2:** Rate of hospitalization per 100 Emergency Department visits by state and by payer. characteristics of California, Florida and the United States in 2009 and 2014.
