# Supplementary material for: Affordable Care Act and healthcare delivery: A comparison of California and Florida hospitals and emergency departments
Source: PLoS One. 2017 Aug 3;12(8):e0182346. doi: 10.1371/journal.pone.0182346 (PMC5542622; doi:10.1371/journal.pone.0182346)
Supplement: S3 Table — Incidence per 1,000 state residents is listed in parentheses. (DOCX) [file pone.0182346.s003.docx]

| **Emergency Department Visit and Hospitalization Trends by State and by Payer** | | | | | | | | | | | | |
| --- | --- | --- | --- | --- | --- | --- | --- | --- | --- | --- | --- | --- |
| **PAYER** | **2009** | **2010** | **2011** | **2012** | **2013** | **2014** | **2009** | **2010** | **2011** | **2012** | **2013** | **2014** |
| **California Emergency Department Visits** | | | | | | | **Florida Emergency Department Visits** | | | | | |
| **Medicare** | 6.0 | 6.2 | 6.6 | 6.9 | 7.1 | 7.3 | 9.4 | 9.9 | 10.5 | 11.0 | 11.1 | 11.8 |
| **Medicaid** | 8.4 | 8.4 | 8.6 | 8.8 | 9.4 | 12.5 | 10.7 | 12.0 | 12.5 | 13.7 | 14.1 | 14.9 |
| **Private Insurance** | 10.4 | 9.6 | 9.8 | 9.9 | 9.5 | 9.7 | 11.2 | 9.9 | 9.9 | 10.2 | 10.3 | 11.5 |
| **Self Pay** | 4.8 | 4.8 | 4.8 | 4.8 | 4.8 | 3.6 | 8.6 | 8.5 | 8.5 | 8.7 | 8.7 | 8.2 |
| **Other** | 2.0 | 2.0 | 2.0 | 2.1 | 2.2 | 1.3 | 2.1 | 2.1 | 2.2 | 2.3 | 2.2 | 2.2 |
| **Total** | 31.6 | 31.0 | 31.7 | 32.6 | 33.1 | 34.5 | 43.3 | 43.4 | 44.5 | 46.7 | 47.2 | 49.4 |
| **California Hospitalizations** | | | | | | | **Florida Hospitalizations** | | | | | |
| **Medicare** | 3.4 | 3.4 | 3.4 | 3.3 | 3.3 | 3.2 | 5.9 | 6.2 | 6.2 | 6.2 | 6.1 | 6.3 |
| **Medicaid** | 2.8 | 2.8 | 2.7 | 2.7 | 2.6 | 3.0 | 2.7 | 2.8 | 2.8 | 2.8 | 2.9 | 2.9 |
| **Private Insurance** | 3.7 | 3.5 | 3.3 | 3.2 | 3.0 | 3.0 | 3.7 | 3.4 | 3.2 | 3.1 | 3.0 | 3.1 |
| **Self Pay** | 0.4 | 0.4 | 0.4 | 0.4 | 0.4 | 0.3 | 0.9 | 0.9 | 0.9 | 0.9 | 0.9 | 0.9 |
| **Other** | 0.5 | 0.6 | 0.6 | 0.6 | 0.6 | 0.4 | 0.8 | 0.7 | 0.8 | 0.7 | 0.7 | 0.7 |
| **Total** | 10.8 | 10.6 | 10.4 | 10.2 | 9.9 | 9.8 | 14.0 | 14.0 | 13.9 | 13.8 | 13.6 | 13.8 |

**Supplementary Table 1: A(top):** Emergency Department visits and Hospitalizations per 100 state residents by state and by payer. **B (bottom):** Total Emergency Department visits and Hospitalizations by state and by payer.

| **Emergency Department Visit and Hospitalization Trends by State and by Payer** | | | | | | | | | | | | |
| --- | --- | --- | --- | --- | --- | --- | --- | --- | --- | --- | --- | --- |
| **PAYER** | **2009** | **2010** | **2011** | **2012** | **2013** | **2014** | **2009** | **2010** | **2011** | **2012** | **2013** | **2014** |
| **California Emergency Department Visits** | | | | | | | **Florida Emergency Department Visits** | | | | | |
| **Medicare** | 2,217,140 | 2,332,927 | 2,473,023 | 2,642,161 | 2,728,855 | 2,838,797 | 1,748,698 | 1,868,829 | 2,000,635 | 2,125,346 | 2,173,574 | 2,343,541 |
| **Medicaid** | 3,115,537 | 3,118,654 | 3,231,593 | 3,362,590 | 3,628,927 | 4,856,934 | 1,997,143 | 2,260,345 | 2,388,890 | 2,648,108 | 2,763,828 | 2,973,816 |
| **Private Insurance** | 3,834,213 | 3,599,506 | 3,684,832 | 3,749,350 | 3,635,666 | 3,781,370 | 2,082,951 | 1,874,506 | 1,900,283 | 1,977,731 | 2,012,948 | 2,278,893 |
| **Self-Pay** | 1,767,439 | 1,781,045 | 1,819,989 | 1,819,954 | 1,833,279 | 1,379,210 | 1,610,149 | 1,604,317 | 1,616,602 | 1,684,634 | 1,704,572 | 1,640,720 |
| **Other** | 726,611 | 738,548 | 757,436 | 796,751 | 858,150 | 501,686 | 396,534 | 400,773 | 427,295 | 436,474 | 425,740 | 441,036 |
| **Total** | 11,662,739 | 11,572,492 | 11,969,359 | 12,406,843 | 12,717,924 | 13,379,786 | 8,078,774 | 8,181,912 | 8,507,682 | 9,045,339 | 9,249,759 | 9,828,338 |
| **California Hospitalizations** | | | | | | | **Florida Hospitalizations** | | | | | |
| **Medicare** | 1256097 | 1286035 | 1285300 | 1267634 | 1257843 | 1227999 | 1101507 | 1161621 | 1192227 | 1206474 | 1203870 | 1244637 |
| **Medicaid** | 1036376 | 1035387 | 1022199 | 1013248 | 1000269 | 1167930 | 504010 | 528998 | 539651 | 550586 | 560427 | 567162 |
| **Private Insurance** | 1351040 | 1288685 | 1257356 | 1222199 | 1163669 | 1148441 | 694883 | 640866 | 608698 | 596129 | 587620 | 621590 |
| **Self-Pay** | 139984 | 150877 | 153434 | 156298 | 153481 | 113516 | 161737 | 167585 | 169702 | 177532 | 181868 | 171059 |
| **Other** | 201371 | 209681 | 214567 | 232392 | 231649 | 136373 | 144027 | 141022 | 145971 | 139799 | 139703 | 137536 |
| **Total** | 3,985,166 | 3,970,921 | 3,933,239 | 3,891,771 | 3,806,911 | 3,794,259 | 2,606,164 | 2,640,092 | 2,656,249 | 2,670,520 | 2,673,488 | 2,741,984 |

| **Rate of Hospitalization from Emergency Department by State and by Payer** | | | | | | | | | | | | |
| --- | --- | --- | --- | --- | --- | --- | --- | --- | --- | --- | --- | --- |
| **PAYER** | **2009** | **2010** | **2011** | **2012** | **2013** | **2014** | **2009** | **2010** | **2011** | **2012** | **2013** | **2014** |
| **California Emergency Department Visits** | | | | | | | **Florida Emergency Department Visits** | | | | | |
| **Medicare** | 38.5 | 38.3 | 37.0 | 34.5 | 33.6 | 31.8 | 45.9 | 45.5 | 44.1 | 42.8 | 42.2 | 41.2 |
| **Medicaid** | 12.4 | 12.8 | 12.5 | 12.2 | 11.4 | 11.6 | 11.9 | 11.8 | 11.7 | 11.0 | 10.7 | 10.2 |
| **Private Insurance** | 12.0 | 12.3 | 11.8 | 11.5 | 11.3 | 10.7 | 15.6 | 16.1 | 15.4 | 14.9 | 14.6 | 14.1 |
| **Self Pay** | 6.6 | 7.1 | 7.0 | 6.6 | 6.1 | 4.6 | 7.9 | 8.9 | 8.8 | 8.6 | 8.9 | 9.0 |
| **Other** | 14.7 | 15.0 | 15.1 | 15.6 | 15.4 | 11.3 | 25.3 | 23.8 | 23.4 | 21.6 | 22.1 | 21.2 |
| **Total** | 16.5 | 17.1 | 16.7 | 16.1 | 15.7 | 14.9 | 20.3 | 20.7 | 20.3 | 19.6 | 19.3 | 18.9 |

**Supplementary Table 2:** Rate of hospitalization per 100 Emergency Department visits by state and by payer. characteristics of California, Florida and the United States in 2009 and 2014.

| **Table 2: ED Visits and Hospitalizations by State and by Clinical Classification Software Multi-Level Categories** | | | | | | | | | | | |
| --- | --- | --- | --- | --- | --- | --- | --- | --- | --- | --- | --- |
| **2009** | **2010** | **2011** | **2012** | **2013** | **2014** | **2009** | **2010** | **2011** | **2012** | **2013** | **2014** |
| **California** | | | | | | **Florida** | | | | | |
| 17.1 Symptoms; signs; and ill-defined conditions (32.3) | 17.1 Symptoms; signs; and ill-defined conditions (31.1) | 17.1 Symptoms; signs; and ill-defined conditions (32.5) | 17.1 Symptoms; signs; and ill-defined conditions (33.9) | 17.1 Symptoms; signs; and ill-defined conditions (35.0) | 17.1 Symptoms; signs; and ill-defined conditions (36.1) | 17.1 Symptoms; signs; and ill-defined conditions (40.7) | 17.1 Symptoms; signs; and ill-defined conditions (41.1) | 17.1 Symptoms; signs; and ill-defined conditions (42.5) | 17.1 Symptoms; signs; and ill-defined conditions (45.6) | 17.1 Symptoms; signs; and ill-defined conditions (45.2) | 17.1 Symptoms; signs; and ill-defined conditions (47.9) |
| 8.1 Respiratory infections (27.6) | 8.1 Respiratory infections (22.3) | 8.1 Respiratory infections (22.9) | 8.1 Respiratory infections (21.0) | 8.1 Respiratory infections (23.4) | 8.1 Respiratory infections (23.0) | 8.1 Respiratory infections (38.7) | 8.1 Respiratory infections (31.8) | 8.1 Respiratory infections (32.2) | 8.1 Respiratory infections (36.2) | 8.1 Respiratory infections (38.4) | 8.1 Respiratory infections (39.9) |
| 7.2 Diseases of the heart (19.1) | 7.2 Diseases of the heart (19.2) | 7.2 Diseases of the heart (19.7) | 7.2 Diseases of the heart (20.6) | 7.2 Diseases of the heart (20.1) | 7.2 Diseases of the heart (20.5) | 7.2 Diseases of the heart (28.5) | 7.2 Diseases of the heart (29.6) | 7.2 Diseases of the heart (29.7) | 7.2 Diseases of the heart (30.8) | 7.2 Diseases of the heart (30.1) | 7.2 Diseases of the heart (31.6) |
| 16.6 Open wounds (14.0) | 16.6 Open wounds (13.7) | 10.1 Diseases of the urinary system (13.8) | 10.1 Diseases of the urinary system (14.5) | 10.1 Diseases of the urinary system (14.8) | 10.1 Diseases of the urinary system (15.9) | 10.1 Diseases of the urinary system (19) | 10.1 Diseases of the urinary system (19.7) | 10.1 Diseases of the urinary system (21.1) | 10.1 Diseases of the urinary system (22.1) | 10.1 Diseases of the urinary system (22.6) | 10.1 Diseases of the urinary system (23.9) |
| 10.1 Diseases of the urinary system (13.2) | 10.1 Diseases of the urinary system (13.2) | 16.6 Open wounds (13.7) | 16.6 Open wounds (13.7) | 16.6 Open wounds (13.3) | 16.6 Open wounds (13.4) | 16.7 Sprains and strains (18.7) | 16.7 Sprains and strains (19.3) | 16.7 Sprains and strains (20.0) | 16.7 Sprains and strains (20.0) | 16.7 Sprains and strains (19.1) | 16.7 Sprains and strains (19.4) |
| 16.8 Superficial injury; contusion (12.0) | 16.8 Superficial injury; contusion (11.7) | 16.8 Superficial injury; contusion (11.7) | 16.8 Superficial injury; contusion (12.1) | 16.8 Superficial injury; contusion (12.0) | 16.8 Superficial injury; contusion (12.6) | 16.8 Superficial injury; contusion (18.6) | 16.8 Superficial injury; contusion (18.4) | 16.8 Superficial injury; contusion (18.6) | 16.8 Superficial injury; contusion (18.8) | 16.8 Superficial injury; contusion (18.4) | 16.8 Superficial injury; contusion (18.7) |
| 16.7 Sprains and strains (11.4) | 16.7 Sprains and strains (11.4) | 16.7 Sprains and strains (11.5) | 16.7 Sprains and strains (11.9) | 16.7 Sprains and strains (11.8) | 16.7 Sprains and strains (12.4) | 16.6 Open wounds (17.1) | 16.6 Open wounds (16.7) | 16.6 Open wounds (16.8) | 16.6 Open wounds (16.9) | 16.6 Open wounds (16.6) | 16.6 Open wounds (16.5) |
| 16.2 Fractures (10.3) | 16.2 Fractures (10.2) | 16.2 Fractures (10.3) | 16.2 Fractures (10.3) | 16.2 Fractures (10.4) | 16.2 Fractures (10.7) | 6.8 Ear conditions (13.4) | 6.8 Ear conditions (13.4) | 6.8 Ear conditions (13.6) | 6.8 Ear conditions (14.1) | 6.8 Ear conditions (13.8) | 13.3 Spondylosis; intervertebral disc disorders; other back problems (14.6) |
| 6.8 Ear conditions (9.7) | 6.8 Ear conditions (9.5) | 6.8 Ear conditions (9.4) | 6.8 Ear conditions (9.3) | 6.8 Ear conditions (9.5) | 6.8 Ear conditions (9.6) | 16.2 Fractures (13.1) | 16.2 Fractures (13.1) | 16.2 Fractures (13.3) | 16.2 Fractures (13.5) | 16.2 Fractures (13.5) | 6.8 Ear conditions (13.9) |
| 17.2 Factors influencing health care (8.0) | 13.3 Spondylosis; intervertebral disc disorders; other back problems (7.5) | 13.3 Spondylosis; intervertebral disc disorders; other back problems (7.7) | 13.3 Spondylosis; intervertebral disc disorders; other back problems (8.2) | 13.3 Spondylosis; intervertebral disc disorders; other back problems (8.3) | 13.3 Spondylosis; intervertebral disc disorders; other back problems (8.9) | 13.3 Spondylosis; intervertebral disc disorders; other back problems (13.1) | 13.3 Spondylosis; intervertebral disc disorders; other back problems (11.5) | 13.3 Spondylosis; intervertebral disc disorders; other back problems (12.2) | 13.3 Spondylosis; intervertebral disc disorders; other back problems (12.8) | 13.3 Spondylosis; intervertebral disc disorders; other back problems (13.3) | 16.2 Fractures (13.8) |
| **California Hospitalizations by Clinical Classification Software Multi-Level Categories** | | | | | | **Florida Hospitalizations by Clinical Classification Software Multi-Level Categories** | | | | | |
| 15.1 Liveborn (13.4) | 15.1 Liveborn (13.4) | 15.1 Liveborn (13.0) | 15.1 Liveborn (12.9) | 15.1 Liveborn (12.5) | 15.1 Liveborn (12.7) | 7.2 Diseases of the heart (17.6) | 7.2 Diseases of the heart (16.9) | 7.2 Diseases of the heart (16) | 7.2 Diseases of the heart (15.6) | 7.2 Diseases of the heart (14.7) | 7.2 Diseases of the heart (14.5) |
| 7.2 Diseases of the heart (9.8) | 7.2 Diseases of the heart (9.8) | 7.2 Diseases of the heart (9.2) | 7.2 Diseases of the heart (8.7) | 7.2 Diseases of the heart (8.2) | 7.2 Diseases of the heart (7.8) | 15.1 Liveborn (11.6) | 15.1 Liveborn (11.1) | 15.1 Liveborn (10.9) | 15.1 Liveborn (10.7) | 15.1 Liveborn (10.6) | 15.1 Liveborn (10.7) |
| 11.3 Complications mainly related to pregnancy (4.4) | 11.3 Complications mainly related to pregnancy (4.4) | 11.3 Complications mainly related to pregnancy (4.5) | 11.3 Complications mainly related to pregnancy (4.5) | 1.1 Bacterial infection (4.6) | 1.1 Bacterial infection (5.2) | 10.1 Diseases of the urinary system (5.0) | 10.1 Diseases of the urinary system (5.1) | 10.1 Diseases of the urinary system (5.6) | 10.1 Diseases of the urinary system (5.7) | 10.1 Diseases of the urinary system (5.7) | 10.1 Diseases of the urinary system (5.8) |
| 11.4 Indications for care in pregnancy; labor; and delivery (3.9) | 11.4 Indications for care in pregnancy; labor; and delivery (3.9) | 1.1 Bacterial infection (3.8) | 1.1 Bacterial infection (4.1) | 11.3 Complications mainly related to pregnancy (4.5) | 11.3 Complications mainly related to pregnancy (4.6) | 8.1 Respiratory infections (4.9) | 8.1 Respiratory infections (4.7) | 8.1 Respiratory infections (4.7) | 8.1 Respiratory infections (4.6) | 5.8 Mood disorders (4.9) | 5.8 Mood disorders (5.1) |
| 8.1 Respiratory infections (3.4) | 8.1 Respiratory infections (3.4) | 11.4 Indications for care in pregnancy; labor; and delivery (3.8) | 11.4 Indications for care in pregnancy; labor; and delivery (3.7) | 11.4 Indications for care in pregnancy; labor; and delivery (3.6) | 11.4 Indications for care in pregnancy; labor; and delivery (3.6) | 9.6 Lower gastrointestinal disorders (4.5) | 9.6 Lower gastrointestinal disorders (4.5) | 5.8 Mood disorders (4.5) | 5.8 Mood disorders (4.6) | 8.1 Respiratory infections (4.7) | 1.1 Bacterial infection (4.6) |
| 1.1 Bacterial infection (3.4) | 1.1 Bacterial infection (3.4) | 8.1 Respiratory infections (3.2) | 9.6 Lower gastrointestinal disorders (3.1) | 5.8 Mood disorders (3.0) | 5.8 Mood disorders (3.0) | 16.10 Complications (4.4) | 16.10 Complications (4.4) | 9.6 Lower gastrointestinal disorders (4.4) | 16.10 Complications (4.4) | 16.10 Complications (4.4) | 8.1 Respiratory infections (4.5) |
| 9.6 Lower gastrointestinal disorders (3.2) | 9.6 Lower gastrointestinal disorders (3.2) | 9.6 Lower gastrointestinal disorders (3.2) | 5.8 Mood disorders (3.1) | 9.6 Lower gastrointestinal disorders (3.0) | 9.6 Lower gastrointestinal disorders (2.9) | 5.8 Mood disorders (4.1) | 5.8 Mood disorders (4.2) | 16.10 Complications (4.4) | 9.6 Lower gastrointestinal disorders (4.4) | 9.6 Lower gastrointestinal disorders (4.3) | 11.3 Complications mainly related to  pregnancy (4.5) |
| 11.5 Complications during labor (3.0) | 11.5 Complications during labor (3.0) | 10.1 Diseases of the urinary system (3.0) | 10.1 Diseases of the urinary system (3.0) | 10.1 Diseases of the urinary system (2.9) | 10.1 Diseases of the urinary system (2.8) | 11.3 Complications mainly related to pregnancy (3.9) | 11.3 Complications mainly related to pregnancy (4.2) | 11.3 Complications mainly related to pregnancy (4.2) | 11.3 Complications mainly related to pregnancy (4.2) | 11.3 Complications mainly related to pregnancy (4.2) | 16.10 Complications (4.3) |
| 10.1 Diseases of the urinary system (3.0) | 10.1 Diseases of the urinary system (3.0) | 5.8 Mood disorders (3.0) | 16.10 Complications (2.9) | 8.1 Respiratory infections (2.8) | 16.10 Complications (2.7) | 16.2 Fractures (3.7) | 16.2 Fractures (3.8) | 16.2 Fractures (3.8) | 16.2 Fractures (3.8) | 1.1 Bacterial infection (3.9) | 9.6 Lower gastrointestinal disorders (4.3) |
| 5.8 Mood disorders (3.0) | 5.8 Mood disorders (3.0) | 16.10 Complications (2.9) | 8.1 Respiratory infections (2.8) | 16.10 Complications (2.8) | 13.2 Non-traumatic joint disorders (2.5) | 7.3 Cerebrovascular disease (3.7) | 7.3 Cerebrovascular disease (3.7) | 7.3 Cerebrovascular disease (3.6) | 7.3 Cerebrovascular disease (3.7) | 16.2 Fractures (3.7) | 16.2 Fractures (3.9) |

**Supplementary Table 3:** Emergency Department (ED) Visits and Hospitalizations sorted by the top 10 most common Clinical Classification Software (CCS) Level-2 categories by state and by year. Incidence per 1,000 state residents is listed in *parentheses*.
